# The Hospital World

**Published:** 1911-09-23

**Authors:** 


### THE HOSPITAL WORLD.

### Death of a well-known Northumberland Practitioner.

The death of the oldest medical practitioner in Northumberland, Dr. Daniel Jackson, J.P., has lately taken place at Hexham. Dr. Jackson's death at the age of seventy-three has been made the subject of many very sympathetic public references. Dr. Jackson was a graduate of Glasgow; he qualified in 1859, taking his M.D. in 1862. He possessed a very large country practice and held many appointments, including those of Certifying Factory Surgeon and Medical Officer of Health for the Hexham Urban District. In public affairs he took a well-balanced interest, and as a Justice of the Peace for the county of Northumberland was recognised as a valuable acquisition to the Bench. He leaves a large family and is succeeded by his son, Dr. John Archibald Jackson, M.D.

### A Nonagenarian Surgeon.

The death is announced, in his ninety-first year, of Mr. James Reid, F.R.C.S.Eng., at his Canterbury home. Mr. Reid became a member of the Royal College of Surgeons as far back as 1843; he was a student of St. Bartholomew's Hospital, and it is interesting to note that he was one of the first occupants of the Warden's Hostel which was inaugurated by Sir James Paget. After spending three years in practice at Dover, he settled in Canterbury, where he devoted his time to surgery. For many years he was surgeon to the Kent and Canterbury Hospital and took a prominent part in obtaining for the cathedral city important sanitary reforms. He was also a student of natural history and became one of the founders of the East Kent Natural History Society.

### A "Liberty Coroner's" Death.

In the person of Mr. Horace Nutt there has passed away an interesting figure and a popular practitioner. Mr. Nutt has been for the past thirty-five years the coroner for the Sherborne Hundred and was, it is said, the last of the "Liberty Coroners," an office which carried with it exceptional justiciary powers. It will be remembered that The Hospital of May 6 last (page 133) contained a special article on "The Ancient Office of Coroner," from the learned and official pen of Dr. F. J. Waldo, H.M. Coroner for the City of London, which attracted wide attention at the time. Mr. Nutt qualified in 1859 at Glasgow, but became a member of the Royal College of Surgeons of England in 1861. For many years he had a large practice in Sherborne, Dorsetshire, where he also held appointments as Medical Officer of Health and Public Vaccinator.

### A Bristol Minister's Hospital Sermons.

Our attention has been called to the fact that whereas the Rev. H. Arnold Thomas was one of the representatives of the Bristol General Hospital at the funeral of the late Mr. H. O. Wills, it may be mentioned that this was peculiarly in keeping, inasmuch as the deceased Mr. Wills' father was for many years an active member of Highbury Congregational Chapel, Cotham, Bristol, under the ministry of the Rev. David Thomas, father of the Rev. H. Arnold Thomas, who succeeded him there. The late Lord Winterstoke, first cousin of the late Mr. H. O. Wills, attended Highbury Chapel for several years, both during the father and son's ministrations. Highbury Chapel has repeatedly responded to hospital appeals. There are Bristol seniors still who can recall the pleading of the Rev. David Thomas on this hospital's behalf. "In one collection this dissenting place of worship has given more than our total collections amount to in a twelvemonth," was the reproach conveyed some quarter of a century ago by the incumbent of a well-attended church in a wealthy part of Bristol.

